# Reversion of antibiotic resistance in multidrug-resistant pathogens using non-antibiotic pharmaceutical benzydamine

**DOI:** 10.1038/s42003-021-02854-z

**Published:** 2021-11-25

**Authors:** Yuan Liu, Ziwen Tong, Jingru Shi, Yuqian Jia, Tian Deng, Zhiqiang Wang

**Affiliations:** 1 College of Veterinary Medicine, Yangzhou University, Yangzhou, 225009 China; 2 Institute of Comparative Medicine, Yangzhou University, Yangzhou, 225009 China; 3 Jiangsu Co-innovation Center for Prevention and Control of Important Animal Infectious Diseases and Zoonoses, Yangzhou University, Yangzhou, 225009 China; 4 Joint International Research Laboratory of Agriculture and Agri-Product Safety, the Ministry of Education of China, Yangzhou University, Yangzhou, 225009 China

**Keywords:** Antimicrobials, Pharmacology

## Abstract

Antimicrobial resistance has been a growing concern that gradually undermines our tradition treatment regimens. The fact that few antibacterial drugs with new scaffolds or targets have been approved in the past two decades aggravates this crisis. Repurposing drugs as potent antibiotic adjuvants offers a cost-effective strategy to mitigate the development of resistance and tackle the increasing infections by multidrug-resistant (MDR) bacteria. Herein, we found that benzydamine, a widely used non‐steroidal anti‐inflammatory drug in clinic, remarkably potentiated broad-spectrum antibiotic-tetracyclines activity against a panel of clinically important pathogens, including MRSA, VRE, MCRPEC and *tet*(X)-positive Gram-negative bacteria. Mechanistic studies showed that benzydamine dissipated membrane potential (▵Ψ) in both Gram-positive and Gram-negative bacteria, which in turn upregulated the transmembrane proton gradient (▵pH) and promoted the uptake of tetracyclines. Additionally, benzydamine exacerbated the oxidative stress by triggering the production of ROS and suppressing GAD system-mediated oxidative defensive. This mode of action explains the great bactericidal activity of the doxycycline-benzydamine combination against different metabolic states of bacteria involve persister cells. As a proof-of-concept, the in vivo efficacy of this drug combination was evidenced in multiple animal infection models. These findings indicate that benzydamine is a potential tetracyclines adjuvant to address life-threatening infections by MDR bacteria.

## Abstract (web summary)

Liu et al. describe the antibiotic adjuvant effect of the FDA approved drug, benzydamine, for the treatment of infections caused by multidrug-resistant bacteria. Mechanistically, benzydamine dissipates membrane potential in both Gram-positive and Gram-negative bacteria, upregulates the transmembrane proton gradient and promotes the uptake of tetracyclines, accounting for its bactericidal activity.

## Introduction

The prevalence of chromosome or plasmid-conferred resistance determinants has severely impaired the efficacy of clinically available antibiotics, rendering the onset of the global antimicrobial resistance crisis^1^. Among these pathogenic bacteria, of particular concern are ESKAPE (Enterococcus, *Staphylococcus aureus*, *Klebsiella pneumoniae*, *Acinetobacter baumannii*, *Pseudomonas aeruginosa*, and *Enterobacter* species), which are responsible for the majority of nosocomial infections worldwide with high morbidity and mortality^2,3^. With the increasing incidence of drug resistance in ESKAPE clinical isolates, bacterial infection-associated diseases are becoming harder to treat. Notably, carbapenems, colistin, and tigecycline are recognized as extremely crucial antibiotics and last options against these drug-resistant bacteria. However, the emergence of carbapenemase^4^, *mcr-1*-encoded phosphoethanolamine transferase^5^, and *tet*(X)-mediated flavin-dependent monooxygenase^6,7^ in bacteria from animal and human source leaves few choices for clinicians from these traditional pipelines. Meanwhile, few novel antibiotic entities with distinct scaffolds or modes of action have been approved for clinical use during the past decades due to the huge scientific and commercial challenges in the development of new drugs^8,9^. There is a dire need to identify alternative strategies to address these infections.

Repurposing drugs as potential antibiotic adjuvants to reverse antibiotic resistance and enhance antibiotic activity represents a simple but effective approach to counter this problem^10–12^. For example, our previous studies have shown that hypoglycemic agent metformin could resensitize *tet*(A)-positive bacteria to tetracyclines through disrupting the functions of efflux pumps^13^. Melatonin, which has been applied for treating sleep disturbances and circadian disorders, potentiated colistin activity against MCR-positive bacteria by enhancing the membrane damage^14^. Anti-HIV agent azidothymidine decreased Tet(X3/X4)-mediated bacterial resistance to tigecycline in *Escherichia coli* via specifically inhibiting DNA synthesis and suppressing resistance enzyme activity^15,16^. In addition, other compounds such as natural products or synthetic drugs have also been demonstrated to enhance the activity of existing antibiotics. The condensed tannins of American cranberry fruit, cranberry proanthocyanidin, increased the activity of a broad range of antibiotic classes against opportunistic pathogens by interfering with intrinsic resistance mechanisms^17^. Synthetic tobramycin−lysine conjugates displayed a synergistic effect with minocycline or rifampicin against clinical multidrug-resistant (MDR) *P. aeruginosa* isolates^18^. Benzydamine is a locally acting nonsteroidal anti-inflammatory drug with local anesthetic and analgesic properties by selectively binding to prostaglandin synthetase^19,20^. Recently, benzydamine was found to inhibit osteoclast differentiation and bone resorption via down-regulating the expression of interleukin-1β^21^. In addition, benzydamine drastically reduced oral mucositis even at doses >50 Gy in head and neck cancer patients^22^. However, the adjuvant potential of benzydamine to existing antibiotics is still not fully understood.

In this study, we characterized the synergistic activity of benzydamine with different classes of antibiotics, and found that it drastically potentiated tetracyclines activity against various MDR pathogens. Importantly, benzydamine dissipated membrane potential (▵Ψ) in both Gram-positive and negative bacteria, which in turn upregulated the transmembrane proton gradient (▵pH) and promoted the uptake of tetracyclines. Meanwhile, benzydamine synergized with doxycycline on killing a spectrum of bacterial pathogens carrying *mec*A, *bla*_MBL_ and/or *mcr* genes, as well as *tet*(X) by triggering oxidative damage. Notably, benzydamine potently rescued the activity of doxycycline in multiple animal infection models infected by MDR MRSA T144 or *E. coli* B2. This study reveals the therapeutic potential of benzydamine as an antibiotic adjuvant for the treatment of infection caused by MDR pathogens.

## Results

### Benzydamine potentiates doxycycline activity in both drug-susceptible and -resistant bacteria

**Fig. 1:**
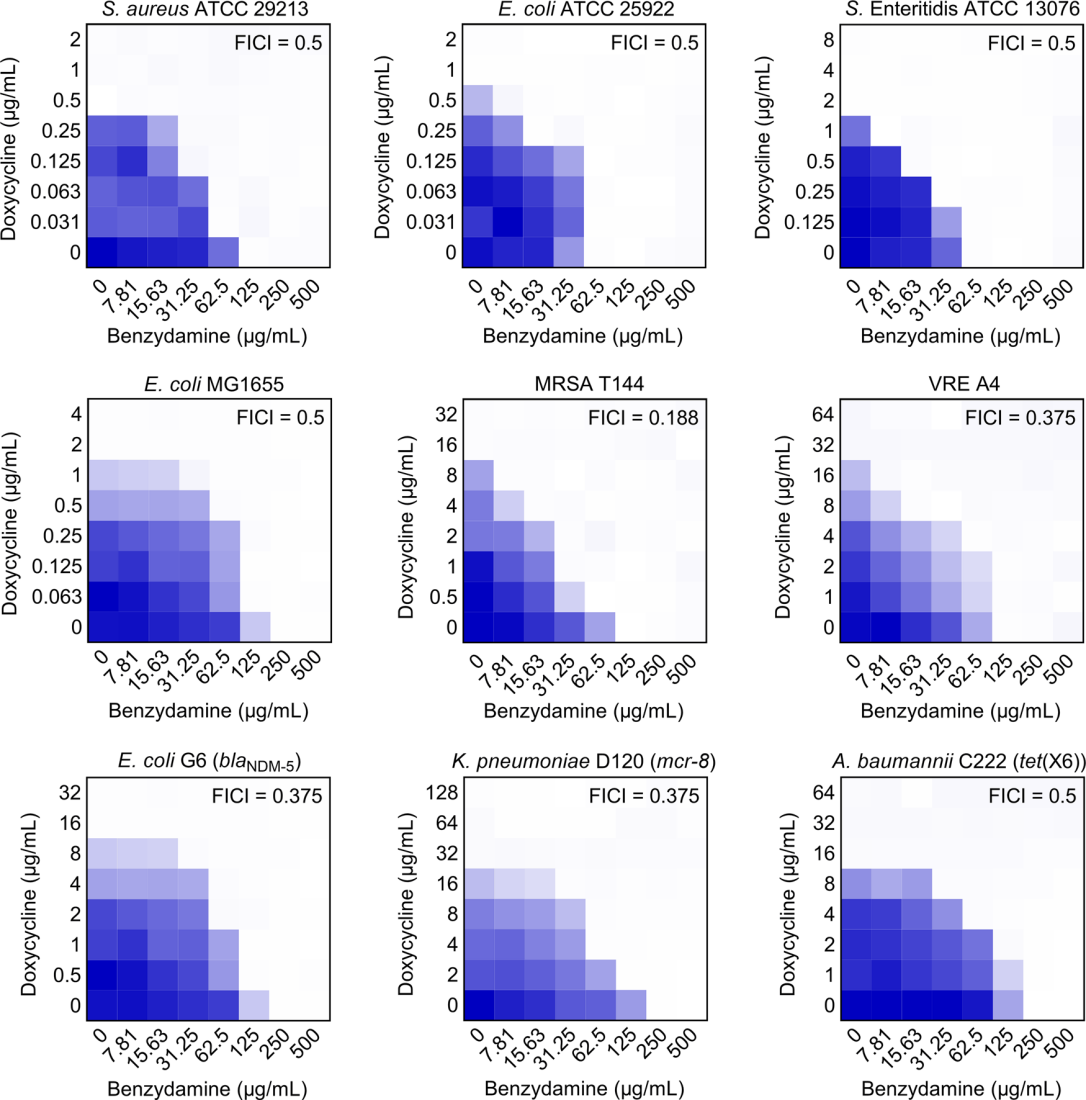
Synergistic activity between benzydamine and doxycycline against drug-susceptible and -resistant bacteria by checkerboard assay, related to Supplementary Table 3. Dark blue regions represent higher cell density. Data represent the mean OD_600_ nm of two biological replicates. Synergy is defined with FIC index ≤0.5.

We first evaluated the synergistic activity of benzydamine with eight classes of antibiotics against MDR *E. coli* B2 using checkerboard broth microdilution assays. Out of these drugs, colistin, ciprofloxacin, and doxycycline showed synergistic activity with benzydamine, whereas kanamycin displayed an antagonistic effect with benzydamine (Supplementary Fig. 1 and Supplementary Table 2). Remarkably, the combination of benzydamine and doxycycline possessed the highest synergistic effect (FIC index (FICI) = 0.188), which enabled the MIC value of doxycycline to decrease from 32 to 2 μg/mL (16-fold). We further tested the potentiation of benzydamine to other tetracyclines, including tetracycline, oxytetracycline, minocycline, and tigecycline. As expected, their antibacterial activity was all substantially improved in the presence of benzydamine (Supplementary Table 2). Subsequently, the checkerboard assays were performed in both drug-susceptible and -resistant bacteria. Interestingly, the combination of benzydamine and doxycycline showed synergistic effect in all test bacteria, including hard-to-treat pathogenic bacteria methicillin-resistant *Staphylococcus aureus* (MRSA) T144 (FICI = 0.188), vancomycin-resistant enterococci (VRE) A4 (FICI = 0.375), *bla*_NDM-5_-positive *E. coli* G6 (FICI = 0.375), *mcr-1*-carrying *K. pneumoniae* D120 (FICI = 0.375) and *tet*(X6)-positive *A. baumannii* C222 (FICI = 0.5). In detail, the MIC values of doxycycline in drug-susceptible bacteria (including *S. aureus* ATCC 29213, *E. coli* ATCC 25922, S. Enteritidis ATCC 13076, and *E. coli* MG1655) were all lower than 2 μg/mL. And in the presence of 0.25× MIC of benzydamine, the MICs of doxycycline were decreased by 4-fold. In contrast, the MICs of doxycycline in drug-resistant pathogens ranged from 16 to 32 μg/mL, which dropped sharply to 1–8 μg/mL at 0.25× MIC of benzydamine, corresponding 4–16-fold potentiating. Overall, this combination displayed a higher synergistic effect on drug-resistant bacteria than -susceptible bacteria, suggesting that its activity is also related to the inhibition of resistance determinants (Fig. 1 and Supplementary Table 3).

Next, we assessed whether the synergistic activity of this combination would result in increased toxicity, including hemolytic activity on mammals RBCs and in vivo toxicity in mice^23^. Surprisingly, no detectable toxicity in hemolysis rate, body weight, and blood biochemical analysis was found in the benzydamine–doxycycline combination treatment (Supplementary Figs. 2 and3). These data suggest that benzydamine is a safe and potent antibiotic adjuvant to tetracyclines.

### Benzydamine dissipates the electric potential (ΔΨ) component of the proton motive force and promotes the uptake of doxycycline

**Fig. 2:**
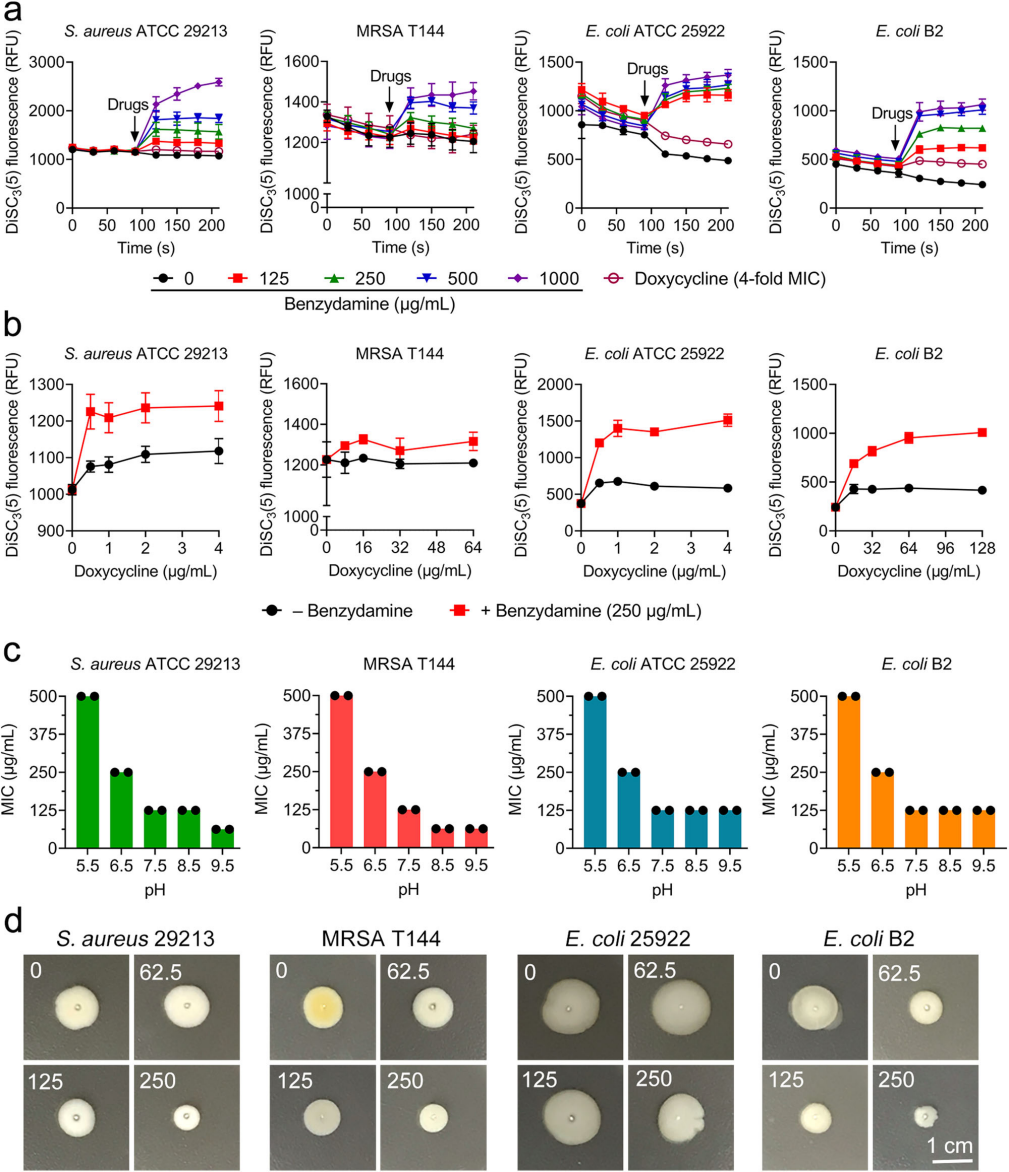
Benzydamine disrupts proton motive force (PMF) in both Gram-positive and Gram-negative bacteria. **a** Benzydamine dissipates membrane potential in bacteria. Fluorescence intensity of DiSC_3_(5) in *S. aureus* ATCC 29213, MRSA T144, *E. coli* ATCC 25922, and *E. coli* B2 after treatment with increasing concentrations of benzylamine and doxycycline (fourfold MIC) was monitored. Drugs were added into DiSC_3_(5)-probed cells at 90 s. **b** Combination of doxycycline and benzydamine (250 μg/mL) displays increased disruption on membrane potential compared with doxycycline alone. **c** Decreased MIC values of benzydamine against four strains in an alkaline environment. ▵Ψ becomes the main component of PMF as the external pH is shifted to an alkaline environment. **d** Benzydamine inhibits the swimming motility of four bacterial strains. Overnight cultures were standardized to OD_600_ nm of 0.5, and inoculated on 0.3% agar plates for 48 h at 37 °C. Scar bar, 1 cm.

Our prior results have shown that benzydamine is a universal adjuvant to tetracycline antibiotics in all tested strains, but antagonizes kanamycin activity in *E. coli* B2. We next assessed the interaction of benzydamine and kanamycin in a panel of bacteria. As a consequence, a remarkable antagonism effect was found in all bacteria (FICI > 2.0, Supplementary Fig. 1 and 4). The opposite effect of benzydamine in combination with doxycycline or kanamycin inspired us to speculate that the mechanism of action of benzydamine may be directly related to the destruction of the bacterial proton motive force (PMF)^24^. In bacteria, the transmembrane transfer of proton H^+^ by the respiratory chain results in an electrochemical gradient, named PMF. It consists of two parts, electric potential (ΔΨ) and transmembrane proton gradient (ΔpH)^25^. Damage to one will be compensated by increasing another to achieve dynamic balance^26^. Previous studies have indicated that the uptake of tetracyclines by bacterial cells depends on ΔpH, whereas aminoglycosides utilize the ΔΨ component for transport, therefore, we are concerned that benzydamine might target the ΔΨ component of bacterial PMF. To test our hypothesis, a fluorescent probe 3,3'-dipropylthiadicarbocyanine iodide (DiSC_3_(5))^27^ was used to assess membrane potential changes induced by doxycycline, benzydamine alone, or their combination in four representative strains (*S. aureus* ATCC 29213, MRSA T144, *E. coli* ATCC 25922 and *E. coli* B2; two Gram-positive and two Gram-negative bacteria). Consequently, no obvious fluorescence change was observed in the doxycycline treatment group. In contrast, benzydamine treatment resulted in rapid disruption of electric potential in a dose-dependent manner (Fig. 2a). Next, we measured the fluorescence with one to fourfold MIC of doxycycline or combination with 250 μg/mL of benzydamine. Benzydamine plus doxycycline indeed resulted in increased fluorescence compared with doxycycline alone (Fig. 2b), suggesting that benzydamine is definitely a potential dissipator of ΔΨ. The extracellular pH values are also related to the PMF. A previous study demonstrated that the antibacterial activity of the dissipater of ΔΨ will be strengthened when the extracellular pH changed to the alkaline values^28^. Consistent with the membrane potential results, the MICs of benzydamine were reduced by four to eightfold when the pH changed from 5.5 to 9.5 in both Gram-negative bacteria and Gram-positive bacteria (Fig. 2c). An intact PMF is required for the bacterial function in flagellar secretion, thus, we next examined the integrity of PMF through swimming motility experiments^29^. Exposure of four strains to sub-inhibitory concentrations of benzydamine drastically decreased bacterial motility (Fig. 2d), suggesting the impaired PMF in benzydamine-treated bacterial cells. These evidence demonstrates that benzydamine disrupts the PMF by targeting ΔΨ component.

**Fig. 3:**
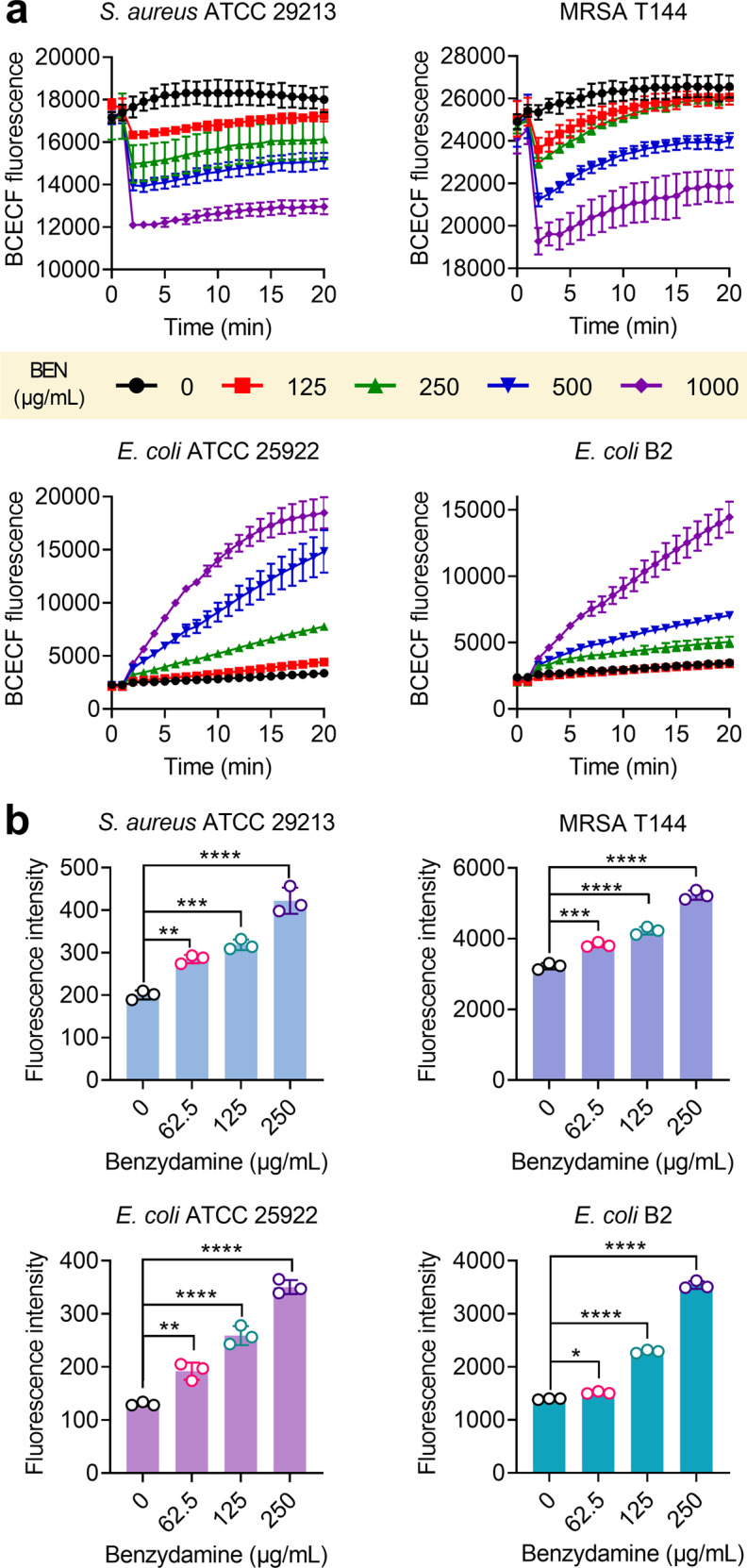
Benzydamine upregulates ▵pH and promotes the intracellular accumulation of doxycycline. **a** Upregulation of ▵pH in BCECF-AM-labeled bacterial cells after exposure to varying concentrations of benzydamine. In Gram-positive bacteria, benzydamine decreases fluorescence and the cytoplasmic pH. In contrast, benzydamine increases fluorescence and the cytoplasmic pH in Gram-negative bacteria (*E. coli* ATCC 25922 and *E. coli* B2). **b** Benzydamine supplementation dose-dependently promotes the intracellular accumulation of doxycycline in bacteria. Intracellular antibiotic content was determined by monitoring the fluorescence of doxycycline (excitation wavelength, 405 nm; emission wavelength, 535 nm). All data from three biological replicates were presented as mean ± SD, and the significance was determined by non-parametric one-way ANOVA (*P < 0.05, **P < 0.01, ***P < 0.001, ****P < 0.0001).

After showing that benzydamine selectively disrupted the ΔΨ, we set out to test whether ΔpH will be compensatorily upregulated. A membrane-permeable fluorescent probe termed BCECF-AM^30^ was used to monitor intracellular pH changes in four strains. Interestingly, benzydamine led to the acidification of the cytoplasm in Gram-positive bacteria, but alkalization of cytoplasm in Gram-negative bacteria (Fig. 3a). Nevertheless, both these actions triggered the upregulation of ΔpH in bacteria. Given that the increasing ΔpH may contribute to the uptake of tetracyclines, thus we next determined the intracellular doxycycline accumulation after exposure to varying concentrations of benzydamine^31^. As expected, benzydamine supplementation remarkably enhanced the content of doxycycline in four strains (Fig. 3b). Tetracyclines act by specifically binding to the 30S subunit of the ribosome, thus inhibiting bacterial protein synthesis^32^. Therefore, the uptake and accumulation of tetracyclines are of importance for their antibacterial activity. Collectively, our results indicate that benzydamine dissipates the ΔΨ, in turn, upregulates the ΔpH, thereby promoting the uptake of tetracyclines.

### Doxycycline plus benzydamine is bactericidal against MDR bacteria and biofilm-producing bacteria

**Fig. 4:**
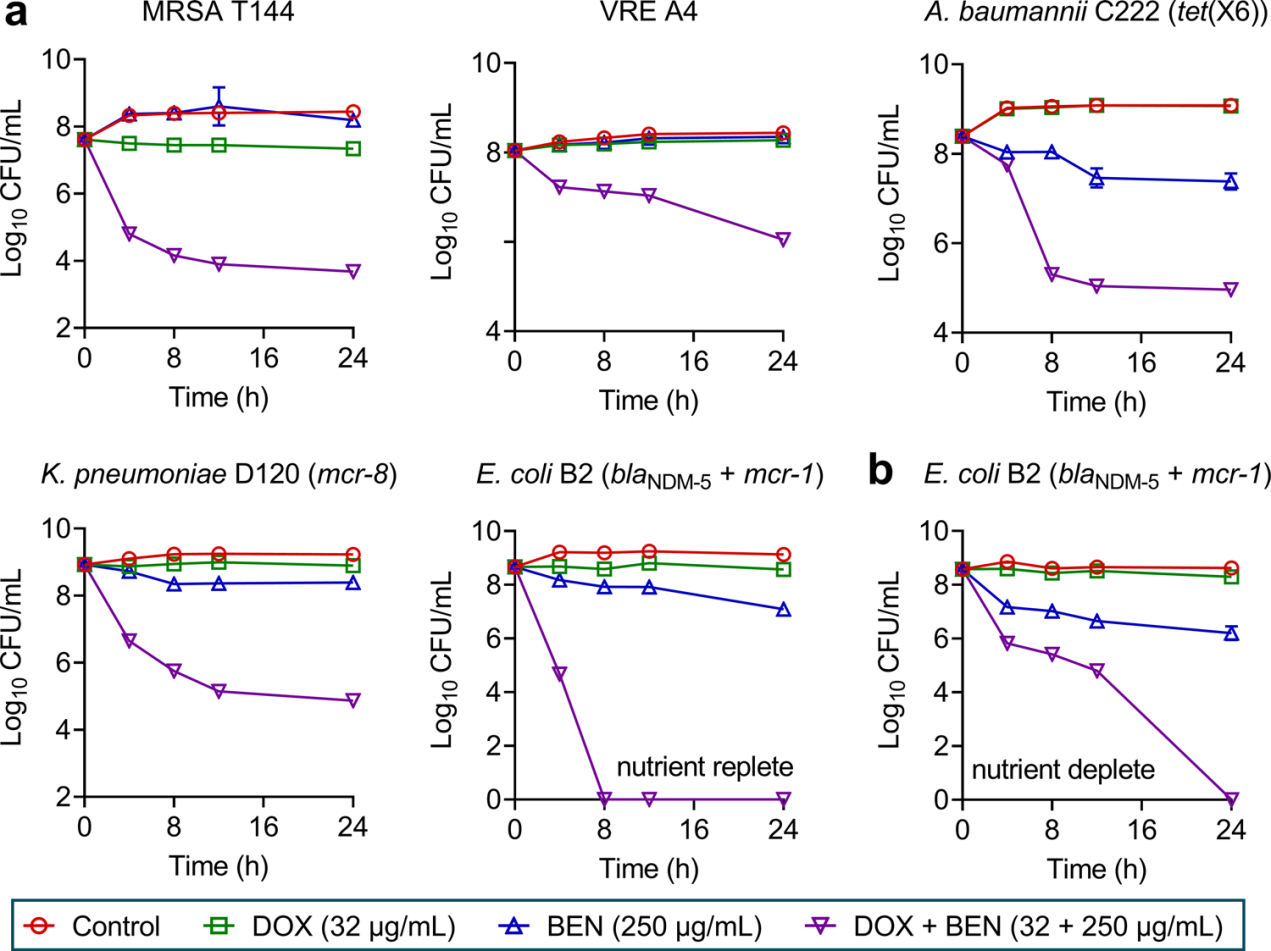
Combination of doxycycline and benzydamine displays bactericidal activity against various drug-resistant bacteria. **a** Killing activity of doxycycline plus benzydamine in LB media against MDR Gram-positive bacteria (MRSA T144 and VRE A4) and Gram-negative bacteria (*A. baumannii* C222, *K. pneumoniae* D120, and *E. coli* B2). **b** Time-killing curves of doxycycline, benzydamine alone, or their combination in PBS against *E. coli* B2. The initial cell density is about 10^8^ CFU/mL. All data were performed from three biological replicates and shown as mean ± SD.

It has been widely acknowledged that tetracyclines belong to bacteriostatic antibiotics. We reasoned whether the benzydamine–doxycycline combination would possess bactericidal activity, which would markedly extend its therapeutic potential. To test this hypothesis, we performed time-killing experiments on various MDR pathogens. Impressively, a direct synergistic bactericidal effect was observed in rich growth conditions (Fig. 4a). Specifically, either 32 μg/mL doxycycline or 250 μg/mL benzydamine showed slight bactericidal activities. In comparison, the combination of doxycycline plus benzydamine (32 + 250 μg/mL) exhibited excellent bactericidal activity, especially for *E. coli* B2. Besides, to determine whether benzydamine has the potency to combat metabolically repressed and non-replicating cells, we tested the bactericidal activity of this drug combination in the nutrient-free buffer. Remarkably, this combination retained potent bactericidal activity against *E. coli* B2 (Fig. 4b).

The formation of antibiotic-tolerant biofilms greatly affects the efficacy of antibiotics^33,34^. To explore whether benzydamine supplementation can enhance doxycycline activity against the biofilm-producing bacteria, we performed the formation and eradication of biofilms experiments in the presence or absence of different concentrations of benzydamine. As shown in Supplementary Fig. 5a, b, the combination of benzydamine at 50 μg/mL with sub-MIC of doxycycline significantly inhibited biofilm formation of MRSA T144 and *E. coli* B2. Notably, in the biofilm inhibition assay, the benzydamine plus doxycycline at concentrations of ≤2 μg/mL did not have bacteriostatic activity against two test strains, indicating that the inhibition of biofilm formation at these concentrations was not owing to the effect on bacterial growth. Besides, in the presence of benzydamine, the eradication effect of doxycycline on mature biofilm was markedly enhanced (Supplementary Fig. 5c, d). Taken together, we unexpectedly found that the combination of doxycycline plus benzydamine displays great bactericidal activity against various MDR pathogens in different metabolic states, including metabolically active cells, antibiotic-tolerant cells, and biofilm-producing bacteria.

### Benzydamine aggravates oxidative damage and inhibits the function of MDR efflux pumps

**Fig. 5:**
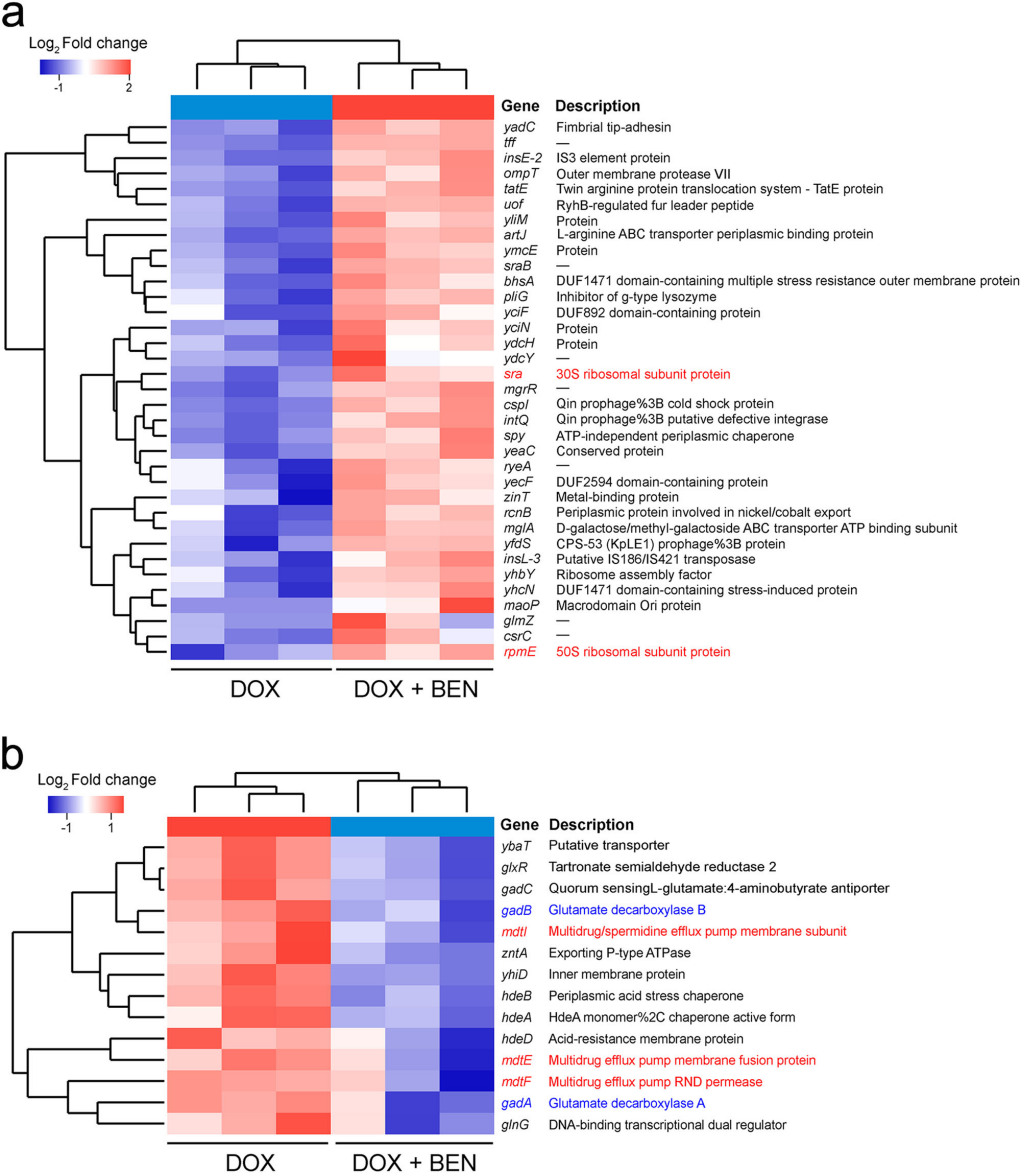
Differentially expressed genes (DEGs) of *E. coli* B2 after treatment with doxycycline plus benzydamine in comparison to doxycycline alone. Significant upregulated (**a**, *P* < 0.05, Log2Fold change ≥ 1) and downregulated DEGs (**b**, *P* < 0.05, Log2Fold change ≤ −1) in combination treatment group compared with doxycycline monotreatment.

Having shown that the synergistic bactericidal activity of the benzydamin–doxycycline combination, we reasoned that benzydamine may trigger other unknown modes of action except for the promotion of doxycycline uptake. To explore the underlying mechanisms, we performed transcription analysis of *E. coli* B2 under treatment with doxycycline or doxycycline plus benzydamine for 4 h. The comparison of treatment with combination to doxycycline alone revealed an upregulation of 35 differentially expressed genes (DEGs) and downregulation of 14 DEGs (Supplementary Fig. 6a). Go annotation analysis showed that these DEGs were involved in biological processes, cellular components, and molecular functions (Supplementary Fig. 6b). KEGG enrichment analysis displayed that these DEGs with increased expression were involved in ribosome synthesis, and DEGs with repressed expression was associated with glutamate metabolism and GABA shunt (Supplementary Fig. 6c, d). Notably, genes with 30S and 50S subunits were upregulated, which may be caused by increased accumulation of doxycycline that inhibits protein synthesis (Fig. 5a). In contrast, multidrug efflux pumps-related genes, glutamate decarboxylase (GAD) system-associated genes, and acid resistance-related genes were obviously decreased in the combination treatment group (Fig. 5b).

**Fig. 6:**
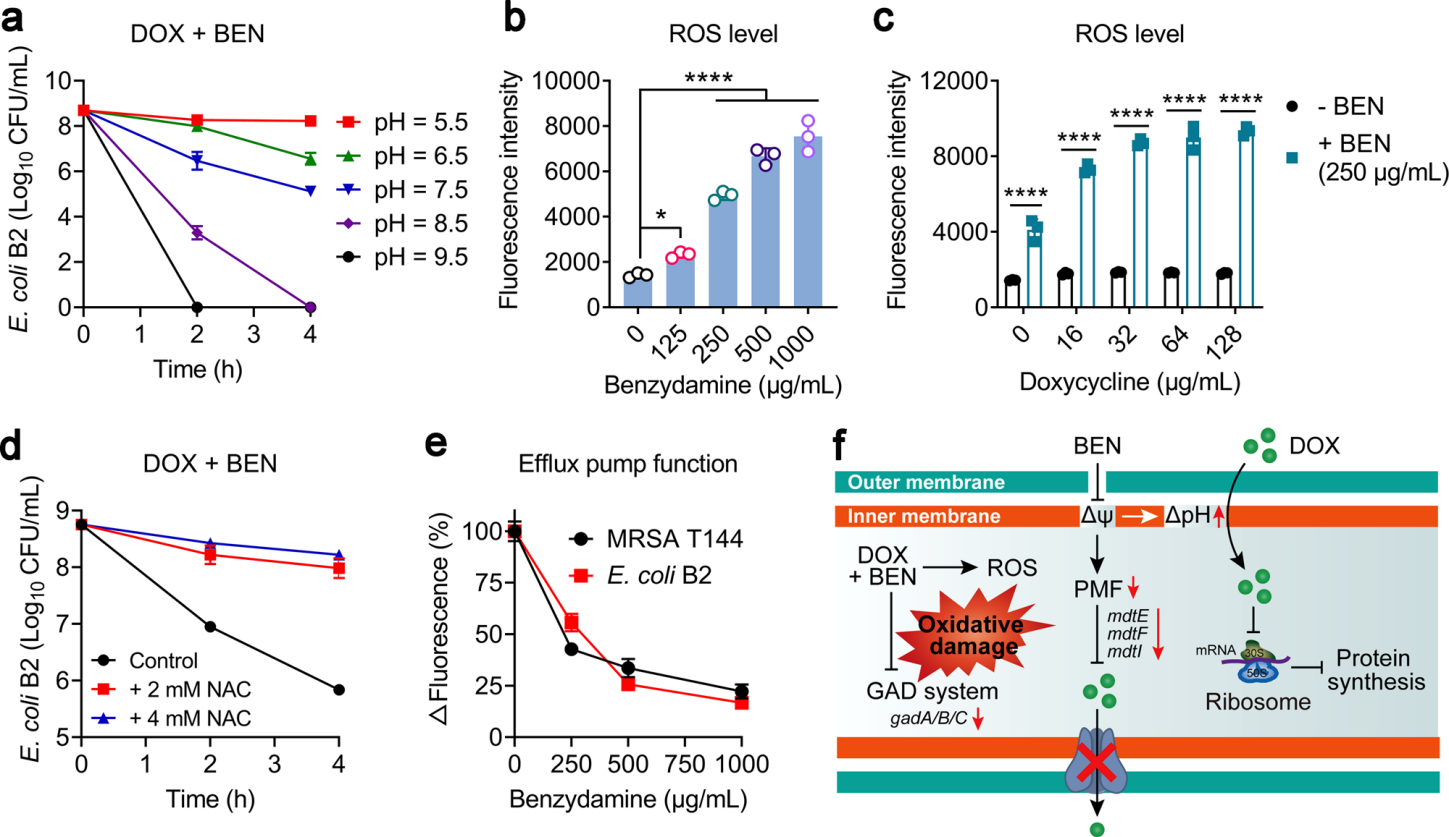
Benzydamine promotes oxidative damage and inhibits the function of the efflux pump in *E. coli*. **a** Time-killing curves of *E. coli* B2 after treatment with the combination of doxycycline and benzydamine in different pH media from 5.5 to 9.5. **b** Benzydamine promotes the production of ROS in a dose-dependent manner. **c** Doxycycline plus benzydamine (250 μg/mL) shows higher ROS generation compared to doxycycline alone. Fluorescence probe 2′,7′-dichlorodihydrofluorescein diacetate (DCFH-DA) was used to monitor the levels of ROS in cells (λexcitation = 488 nm, λemission = 525 nm). All data from three biological replicates were presented as mean ± SD, and the significance was determined by non-parametric one-way ANOVA (*P < 0.05, **P < 0.01, ***P < 0.001, ****P < 0.0001). **d** The addition of ROS scavenger *N*-acetylcysteine weakens the potentiation of benzydamine to doxycycline. **e** Benzydamine drastically impairs the function of efflux pumps in both MRSA T144 and *E. coli* B2. Rhodamine B (λexcitation = 540 nm, λemission = 625 nm) was used to characterize the activity of efflux pumps in bacteria. **f** Schematic illustrations of the potentiating mechanisms of benzydamine to tetracyclines in the fight against drug-resistant pathogens.

Based on the transcription results, we next performed a series of phenotype experiments to elucidate the other functions of benzydamine. First, we evaluated the activity of drug combinations in the different pH growth environments via time-killing experiments. We found that it had almost no bactericidal effect in the acid media, however, this combination showed enhanced bactericidal activity while a change in pH to alkaline values. After treatment for 4 h, the bacteria were all killed in the MHB broth at pH 8.5 and 9.5 (Fig. 6a). These data were in agreement with the previous observations that the antibacterial activity of benzydamine was strengthened in the alkaline conditions and the genes associated with acid resistance were downregulated. Recently, several studies reported that GAD systems, which were downregulated in the combination group, play a critical role in protecting bacteria against oxidative stress^35,36^, we hypothesized that the potentiation of benzydamine to antibiotics may also correlate to enhanced oxidative damage. Thus, we tested the generation of reactive oxygen species (ROS)^37^ in *E. coli* B2 treated by either benzydamine or in combination with doxycycline. Surprisingly, benzydamine markedly promoted the generation of ROS in a dose-dependent manner (Fig. 6b). Meanwhile, the combination treatment showed higher ROS levels compared to doxycycline monotreatment (Fig. 6c). Accordingly, ROS has been recognized as one of the common mechanisms in the antibiotic-mediated killing of bacteria. The over-production of ROS in the benzydamine–doxycycline combination gives an interpretation of their synergistic bactericidal activity. To further verify it, *N*-acetyl-l-cysteine (NAC), a ROS scavenger, was added in time-killing assays. As shown in Fig. 6d, the potentiation of benzydamine to doxycycline was greatly impaired when incubation with 2 or 4 mM NAC. Finally, we used a fluorescent dye rhodamine B to assay the function of the efflux pump in bacteria under the treatment of benzydamine (Fig. 6e). As a result, it showed that the activity of the efflux pump was dose-dependently suppressed by benzydamine, which further promoted the accumulation of doxycycline in MDR bacteria. Collectively, these data together demonstrate that benzydamine enhances oxidative damage via triggering the production of ROS and inhibiting the function of MDR efflux pumps (Fig. 6f).

### Benzydamine rescues doxycycline efficacy in vivo infection models

**Fig. 7:**
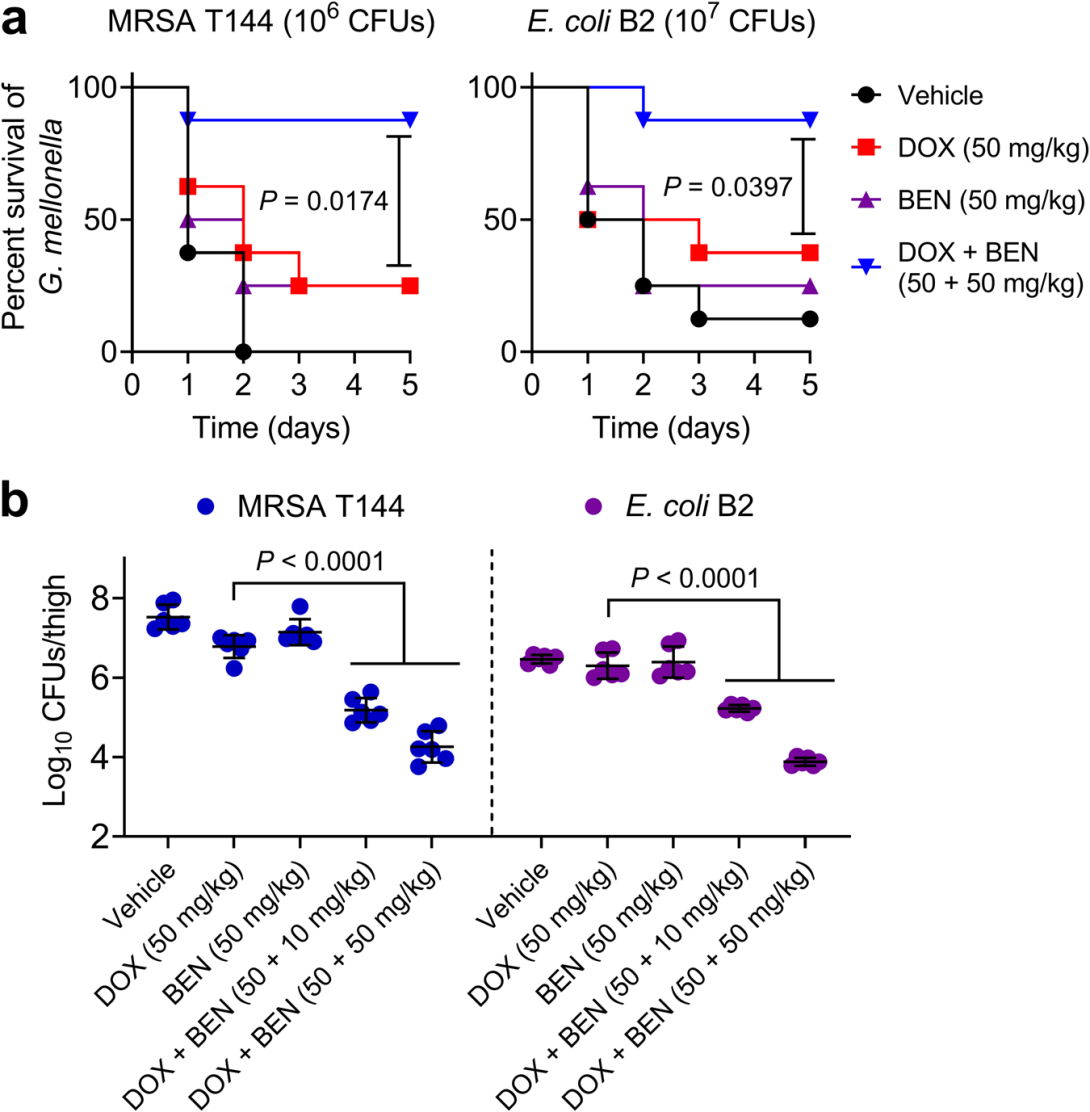
Combination of doxycycline and bezydamine is efficacious in various animal infection models. **a** Survival rates of *Galleria mellonella* (*n* = 8 biologically independent animals per group) infected by MRSA T144 or *E. coli* B2 and then treated with doxycycline (50 mg/kg) or benzydamine (50 mg/kg ) alone or a combination of doxycycline plus benzydamine (50 + 50 mg/kg). *P* values were determined using the two-sided log-rank (Mantel–Cox) test. **b** Combination of doxycycline (50 mg/kg) and benzydamine (50 mg/kg) significantly reduces the thigh bacterial loads of mice (*n* = 6 biologically independent animals per group) infected by MRSA T144 or *E. coli* B2 (10^5^ CFUs per mouse) compared with doxycycline monotherapy (50 mg/kg). *P* values were calculated using a two-sided Mann–Whitney *U* test.

In view of the excellent synergy of doxycycline and benzydamine against MDR pathogens in vitro, we tested whether they have potent activity in *vivo* using two animal infection models infected by MRSA T144 or *E. coli* B2. First, we used *G*. *mellonella* larvae infection models to explore their efficacy in *vivo*. As shown in Fig. 7a, the infected larvae with the combination therapy of doxycycline plus benzydamine (50 + 50 mg/kg) resulted in above 80% survival during 5 days, which was higher than the doxycycline monotreatment (*P* = 0.0174 or 0.0397, corresponding to MRSA T144 and *E. coli* B2, respectively). In addition, the efficacy of this combination therapy in a neutropenic mouse thigh infection model was evaluated (Fig. 7b). Similarly, a single dose of doxycycline plus benzydamine (50 + 10 or 50 + 50 mg/kg) reduced bacterial burden in mice thighs compared with doxycycline alone (*P* < 0.0001). These data demonstrate the benzydamine plus doxycycline has a great synergy effect in vivo.

## Discussion

The occurrence of MDR phenotype in pathogenic bacteria undermines the clinical efficacy of antibiotics and leaves no available options for the treatment of intractable infections^38,39^. Despite many ongoing effects in identifying new classes of antimicrobial agents^40,41^, few antibiotics have been approved for clinical use in the past 20 years. Accordingly, the average cost of research and development of a new drug from discovery to regulatory approval is US$2.6 billion, which takes >10 years, and the successful launch is less than one thousandth^42,43^. As such, alternative strategies are warranted to confront this serious global crisis. Repurposing non-antibacterial drugs as potential antibiotic adjuvants are gaining traction in both the public and private sector^44^. In this study, we revealed the synergistic effect of benzydamine, a widely used non‐steroidal anti‐inflammatory drug in the clinic, in combination with three classes of antibiotics against MDR *E. coli* B2. Most importantly, benzydamine displayed the greatest synergistic activity with tetracyclines, which belong to broad-spectrum antibiotics. For various MDR pathogens, including MRSA, VRE, NDM/MCR/Tet(X)-expressing Gram-negative bacteria, the doxycycline–benzydamine combination showed unprecedented synergistic activity. Biofilm-producing bacteria are important causes of chronic and recurrent bacterial infection but are commonly overlooked in drug discovery. We found that the doxycycline–benzydamine combination is able to prevent the formation of *S. aureus* and *E. coli* biofilm, as well as eradicate the established biofilms. These data further support the notion that benzydamine is a potential antibiotic adjuvant candidate to reverse bacterial resistance. Consistent with our finding, the prior study provided a preliminary observation regarding the antibacterial activity of the benzydamine-tetracyclines combination, whereas the antibacterial spectrum, mechanisms of action, and in vivo potency are not explored^45^.

Bacterial energy metabolism such as PMF has a critical role in cellular activities including material transport, flagellar motility, and ATP synthesis by the F_1_F_0_-ATPase^46^. The disruption of PMF would inhibit the basic functions of bacteria and accelerate its death. Recently, using a deep learning approach, a new broad-spectrum antibiotic termed halicin was identified to selectively destroy the PMF^47^. Molecules I1- I3 and D1-D3, the potential modulators of PMF, showed killing activity against MRSA by preventing electron transport and ATP synthesis^28^. Generally, PMF is comprised of ΔΨ and ΔpH. To maintain the bacterial PMF, dissipation of either component would be compensatory increased by another. In our study, we uncovered that benzydamine dissipated the ΔΨ component of PMF in both Gram-positive and Gram-negative bacteria, in turn, increased the ΔpH, which was critical for the uptake of tetracyclines. These findings were consistent with the previous studies that tetracyclines uptake is driven by ΔpH^48^, whereas aminoglycosides uptake is highly dependent on ΔΨ^49^. In addition, the successful paradigm of daptomycin depolarizing the cytoplasmic membrane provides a proof-of-concept for PMF-targeted antimicrobial agents^50^. These examples suggest that bacterial PMF is a promising target for the development of novel antimicrobial agents and antibiotic adjuvants. Toxicity concerns are critical factors that limit the clinical trials of new drugs^51^. Meaningfully, our experiments tentatively demonstrated that the combination of doxycycline and benzydamine exhibited negligible toxicity in a murine model. Nevertheless, considering the potential side effects of benzydamine in clinic such as nausea and vomiting^52^, more preclinical studies are needed for exploring the therapeutic potential of this particular combination. Moreover, we proposed that the structure optimization of benzydamine and the use of functionalized drug delivery system^53^ may contribute to reducing the toxicity and improving the effectiveness of this drug combination.

Furthermore, the doxycycline–benzydamine combination showed excellent synergistic bactericidal activity for all test MDR isolates, implying that the action of benzydamine is not merely to promote the uptake of tetracyclines. Transcriptomic analysis coupled with phenotype experiments indicated that benzydamine not only triggered the generation of ROS, but also downregulated the GAD system that protects bacteria from oxidative damage. These modes of action resulted in an oxidative burst, which has been proved to be important for antibiotic-mediated killing. In addition, the functions of MDR efflux pumps in bacteria were severely destroyed, partly owing to the dissipation of PMF by benzydamine. It would be interesting to investigate the potential of benzydamine as a new and broad-spectrum inhibitor of MDR efflux pumps.

To conclude, our findings reveal that non‐steroidal anti‐inflammatory drug benzydamine may serve as a potential antibiotic adjuvant to restore the activity of clinically relevant antibiotics particularly tetracyclines against infections caused by MDR pathogens. In addition, the elucidation of modes of action of benzydamine highlights the remarkable potential of PMF downregulators as a feasible adjuvant therapy to tackle the escalating concern of antibiotic resistance.

## Materials and methods

### Bacterial strains and reagents

All strains used in this study were listed in Supplementary Table 1. The bacteria were stored in nutrient broth supplemented with 20% (v/v) glycerol at −80 °C. For experiments, all strains were grown in Mueller-Hinton Broth (MHB) or on LB agar (LBA) plates. Antibiotics were obtained from China Institute of Veterinary Drug Control and other chemical reagents were purchased from Aladdin (Shanghai, China) or TCI (Shanghai, China).

### MIC determinations

The MICs of all antibiotics and benzydamine were determined using the broth dilution method, according to the CLSI 2018 guideline^54^. All drugs were twofold diluted in MHB and equally mixed with bacterial suspensions in a 96-well microtiter plate (Corning, New York, USA). After 16–20 h incubation at 37 °C, the MICs were defined as the lowest concentrations of drugs that no bacteria can be detected.

### Checkerboard analyses and FIC index determination

The fractional inhibitory concentrations (FIC) indices were measured by the checkerboard analyses^55^. In brief, 100 μL of MHB was added into each well of a 96-well plate with an 8 × 8 matrix, then the antibiotics and compounds were twofold diluted along the abscissa and ordinate, respectively. After incubated at 37°C for 18 h with bacterial suspension (10^5^ CFUs/well), the absorbance of each well at 600 nm was determined. The FIC was calculated as the MIC when the compound is used in combination divided by the MIC when it is used alone. The FICI is the sum of the FICs of two compounds, and synergy is defined with the FIC index ≤0.5.

### Hemolysis analysis

Hemolytic activity of doxycycline or in combination with benzydamine was assessed based on a previous study^56^. In brief, 8% of sheep blood cells were equal to volume incubated with 0–256 μg/mL doxycycline alone or in combination with 250 μg/mL benzydamine at 37 °C for 1 h. Phosphate buffer saline (PBS) and double-distilled water were used as a negative and positive control, respectively. The absorbance of released hemoglobin was determined at 576 nm using an Infinite M200 Microplate reader (Tecan, Männedorf, Switzerland). Hemolysis rate (%) was calculated by the result of absorbance of the sample subtracting the negative control divided by the positive control subtracting the negative control.

### In vivo toxicity of benzydamine–doxycycline combination

The in vivo toxicity was evaluated by gavaging a combination of doxycycline plus benzydamine (10 + 10 mg/kg) to female CD-1 mice (*n* = 6 per group). Mice were continuously gavaged for 6 days and body weights were recorded daily. On the seventh day, blood was collected for blood biochemical tests and cell analysis.

### Inhibition of biofilm formation

MRSA T144 and *E. coli* B2 suspensions (1 × 10^7^ CFUs per mL) were exposed to doxycycline solutions (final concentrations ranging from 0.125 to 2 μg/mL) in the presence or absence of 50 μg/mL benzydamine. As a negative control, bacteria were exposed to MHB without drugs. Bacteria were grown for 36 h at 37 °C under static conditions, and then 300 μL PBS was used to remove the planktonic bacteria. Then, 200 μL of methanol was added to fix for 15 min, after that, the fixative was aspirated to air dry and 0.1% crystal violet was added for staining for 15 min. The dye solution was removed and stained-biofilm was washed three times with PBS and dried naturally. At last, crystal violet-stained biofilms were solubilized with 33% glacial acetic acid (100 μL) and incubated at 37 °C for 30 min. Biofilm mass was determined by monitoring the absorbance of the supernatant at 570 nm^57^.

### Biofilm eradication assay

Overnight MRSA T144 and *E. coli* B2 were diluted 1:100 into MHB and incubated at 37 °C with sharking at 200 rpm for 6 h. Subsequently, 100 μL bacterial suspensions were mixed with an equal volume of MHB in 96-well microtitre plate. After 36 h incubation at 37 °C, the planktonic bacteria were removed. Next, biofilms were treated with 32–256 μg/mL doxycycline alone or in combination with 50 μg/mL benzydamine. After 2 h incubation at 37 °C, the remaining cells were dispersed via ultrasonic treatment for 20 min. Finally, the mixed liquor was resuspended in sterile PBS and then the dilutions were plated on LBA plates and incubated overnight at 37 °C.

### Measurement of membrane potential

The membrane potential of *S. aureus* ATCC 29213, MRSA T144, *E. coli* ATCC 25922, and *E. coli* B2 was tested by the fluorescent probe DiSC_3_(5) (Aladdin, Shanghai, China). Bacterial cells were grown to the log phase in MHB, then washed with PBS to an OD_600_ of 0.5 and incubated with DiSC_3_(5) (0.5 × 10^−6^ M) for 30 min. Finally, varying concentrations of benzydamine (10 μL) were added into the 190 μL of DiSC_3_(5)-loaded cells. For all membrane potential experiments, the fluorescence intensity was measured with the excitation wavelength at 622 nm and emission wavelength at 670 nm using a Microplate reader (Tecan, Männedorf, Switzerland).

### Swimming motility experiment

In all, 0.3% (w/v) agar media composed of trypticase peptone (10 g/L), NaCl (10 g/L), and yeast extract (5 g/L) was used to assess bacterial swimming motility^31^. After the medium reached 50 °C, the final concentrations of benzydamine at 0, 31.25, 62.5, 125, and 250 μg/mL were added. A 2 µL volume of *S. aureus* 29213, MRSA T144, *E. coli* 25922, and *E. coli* B2 culture at an OD_600_ of 0.5 was placed in the center of each plate and allowed to stay for 30 min. The plates were placed in a 37 °C incubator for 48 h.

### Measurement of intracellular pH values

Overnight *S. aureus* ATCC 29213, MRSA T144, *E. coli* ATCC 25922, and *E. coli* B2 were resuspended to OD_600_ of 0.5 with PBS, and the final concentration of pH-sensitive fluorescent probe BCECF-AM^30^ (2 × 10^−6^ M for Gram-negative bacteria and 0.5 × 10^−6^ M for Gram-positive bacteria) was added. After incubation for 30 min, four strains were treated with the final concentration of benzydamine (125–1000 μg/mL). The fluorescence intensity was immediately monitored with the excitation wavelength of 488 nm and emission wavelength of 535 nm.

### Uptake of doxycycline

Doxycycline uptake was evaluated by monitoring the fluorescence change of the drug in bacteria^31^. Culture of MRSA T144 and *E. coli* B2 were grown to an OD_600_ of 0.5. Cells were centrifuged at 3500 rpm for 10 min and washed in an equal volume of PBS three times. Subsequently, doxycycline at MIC alone or with a final concentration of benzydamine at 250–1000 μg/mL was added into the 96-wells plates containing cell suspensions at 100 µL/well. An infinite Microplate reader was used to monitor the fluorescence intensity with the excitation wavelength of 405 nm and emission wavelength of 535 nm.

### Measurement of ROS levels

The fluorescence probe 2′,7′-dichlorodihydrofluorescein diacetate (DCFH-DA, 10 μM)^58^ (Beyotime, Shanghai, China) was used to test the levels of ROS in *E. coli* B2 treated by benzydamine, doxycycline, or their combination. After incubation 1 h, the fluorescence intensity was detected with the excitation and emission wavelength of 488 and 525 nm, respectively.

### Efflux pump assay

A fluorescence dye, rhodamine B^59^ has been applied to assay the inhibition of efflux pump of *E. coli* B2 and MRSA T144 treated by benzydamine. Bacterial cells were grown in MHB broth to mid-log phase (OD_600_ = 0.5) at 37 °C with shaking 200 rpm, then the cultures were washed and suspended with PBS. Subsequently, a final concentration of rhodamine B (Aladdin, Shanghai, China) (5 × 10^−6^ M) was added. After incubation at 37 °C for 30 min, the cultures were washed and suspended with PBS containing 1% glucose. Next, probe labeled cells were treated with varying concentrations of benzydamine. After incubation at 37 °C for 30 min, bacterial cells were centrifuged at 6000 rpm at 4 °C for 5 min and the supernatant was collected for fluorescence analysis with the excitation wavelength of 540 nm and emission wavelength of 625 nm.

### Time-dependent killing curves

Overnight culture of *E. coli* B2, *K. pneumoniae* D120 (*mcr-8*), *A. baumannii* C222 (*tet*(X6)), MRSA T144, and VRE A4 were diluted 1/1000 in MHB, and incubated for 4 h at 37 °C with sharking at 200 rpm. Bacteria were then treated with either PBS, doxycycline (16 or 32 μg/mL), or benzydamine (250 μg/mL) alone or their combination. At the time points 0, 4, 8, 12, and 24 h, 100 μL aliquots were removed, centrifuged, and resuspended in sterile PBS, the dilutions were plated on LBA plates, and incubated overnight at 37 °C.

### Transcriptomic analysis

The final concentration of doxycycline (32 μg/mL) alone or in the combination with benzydamine (250 μg/mL) was added to the early exponential-phase *E. coli* B2. After incubation for 4 h, the total RNA of culture was extracted by an EASYspin Plus kit (Aidlab, Beijing, China) and quantified by using a Nanodrop spectrophotometer (Thermo Scientific, MA, USA). Subsequently, sequenced on Hiseq2000 with Truseq SBS Kit v3‐HS (200 cycles) (Illumina) with the read length as 2 × 100 (PE100). Raw sequencing reads were filtrated and mapped against the reference genome of *E. coli* K-12. The FPKM (Fragments Per Kilobase of transcript per Million mapped reads) method was used to identify differentially expressed genes with *p* values ≤0.05 and fold change (FC) values ≥2 (log2 FC ≥ 1 or log2 FC ≤ −1). Differences between these two treatments were studied by Cuffdiff program (http://cufflinks.cbcb.umd.edu/).

### *Galleria mellonella* infection model

*Galleria mellonella* larvae (Huiyude Biotech Company, Tianjin, China) were divided into four groups (*n* = 8 per group) and infected with MRSA T144 (10^6^ CFU_S_) or *E. coli* B2 (10^7^ CFU_S_) suspension. After 1 h post infection, group 1 was subjected to PBS treatment, groups 2 and 3 were treated with doxycycline or benzydamine (50 mg/kg) respectively, group 4 was treated with doxycycline plus benzydamine (50 + 50 mg/kg). Survival rates of *Galleria mellonella* larvae were recorded for 5 days.

### Neutropenic mouse thigh infection model

6–8-week-old female CD-1 mice were obtained from the Comparative Medicine Centre of Yangzhou University (Jiangsu, China). Mice studies were performed in accordance with the relevant guidelines of Jiangsu Laboratory Animal Welfare and Ethical of Jiangsu Administrative Committee of Laboratory Animals (permission number, SYXKSU-2007-0005). The laboratory animal usage license number is SCXK-2017-0044, certified by Jiangsu Association for Science and Technology.

Female CD-1 mice (*n* = 6 per group) were treated with cyclophosphamide with 150 mg/kg in the 4 days before infection, and 100 mg/kg in the 1 day before infection. The exponential-phase MRSA T144 or *E. coli* B2 suspension (100 μL, 10^5^ CFUs per mouse) was injected into the right thighs of mice. After 2 h of infection, a single dose of PBS, doxycycline (50 mg/kg), benzydamine (50 mg/kg), or combinations (50 + 10 mg/kg, 50 + 50 mg/kg) was administered. At 48 h post infection, mice were euthanized due to dislocation of the cervical spine. The right thigh muscle was aseptically resected, homogenized, serially diluted, and plated on LBA to determine bacterial numbers.

### Statistics and reproducibility

Statistical analysis was performed using GraphPad Prism version 8.3.0. All data from at least three biological replicates are shown as mean ± SD. Unless otherwise noted, unpaired *t* test between two groups or one-way analysis of variance among multiple groups were used to calculate *P* values (*P < 0.05, **P < 0.01, ***P < 0.001, and ****P < 0.0001).

### Reporting summary

Further information on research design is available in the Nature Research Reporting Summary linked to this article.

## Supplementary information

Supplementary Information

Description of Additional Supplementary Files

Supplementary Data 1

Reporting Summary

## Data Availability

RNA-sequencing data have been deposited in the National Center for Biotechnology Information (NCBI) Sequence Read Archive (SRA) database (PRJNA766533). Source data for the main figures are provided in Supplementary Data 1. All other data are available from the corresponding authors.
