# Supplementary material for: Reversion of antibiotic resistance in multidrug-resistant pathogens using non-antibiotic pharmaceutical benzydamine
Source: Commun Biol. 2021 Nov 25;4:1328. doi: 10.1038/s42003-021-02854-z (PMC8616900; doi:10.1038/s42003-021-02854-z)
Supplement: Supplementary file 1 — Supplementary Information [file 42003_2021_2854_MOESM1_ESM.pdf]

Supplementary Information

Figures

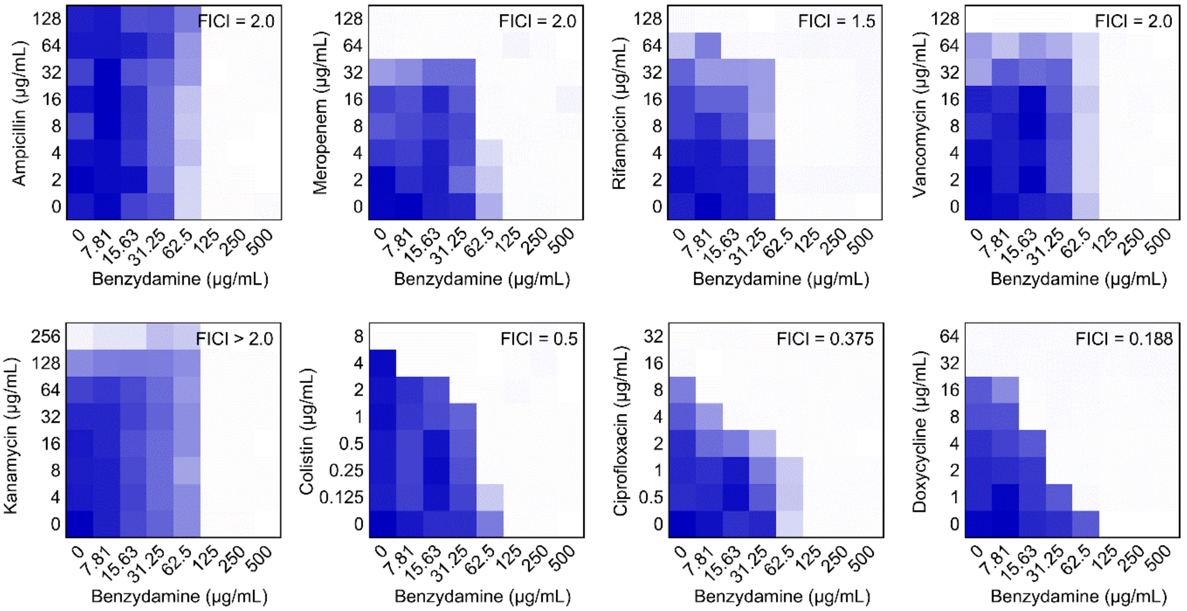

Supplementary Fig. 1 Interaction between benzydamine and multiple classes of antibiotics against *E. coli* B2 by checkerboard assay, related to Supplementary Table 2. Dark blue regions represent higher cell density. Data represent the mean OD (600 nm) of two biological replicates.

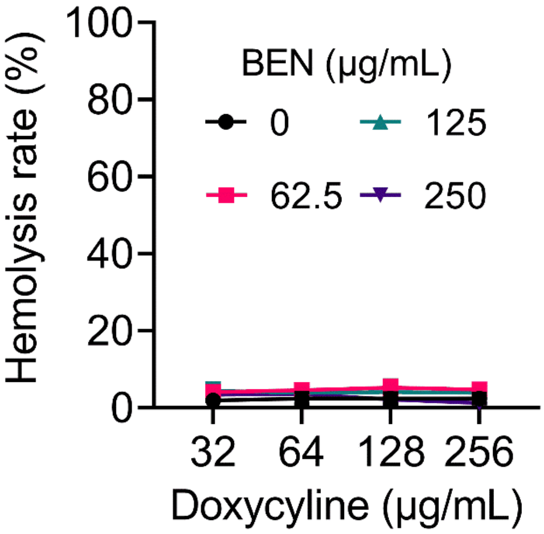

Supplementary Fig. 2 Doxycycline plus benzydamine displays negligible hemolytic activity on mammals' RBCs.

Phosphate buffer saline (PBS) and double-distilled water were used as negative and positive control, respectively.

a

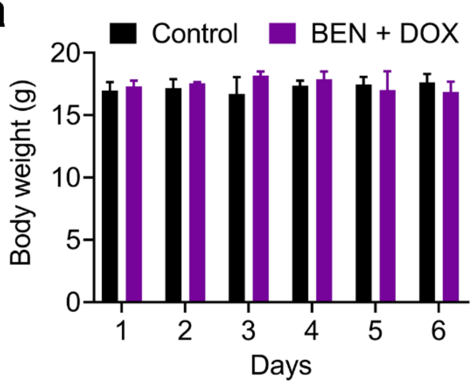

b

Parameters	Control	BEN + DOX
Glucose	5.6	4.9
Urea Nitrogen	9.10	4.1
Ca^{2+}	2.34	2.38
Creatinine	40	45.3
Albumin	30.3	23.1
Phosphorous	1.94	2.18
Glutamate transferase	44.8	45.7

c

Parameters	Unit	Control	BEN + DOX
WBC	10^9 cells/L	5.0	6.2
LYM	10^9 cells/L	3.7	4.1
MON	10^9 cells/L	0.2	0.1
GRAN	10^9 cells/L	1.1	2.0
LYM	%	74.0	64.9
MON	%	3.2	3.2
GRAN	%	22.8	32.8
RBC	10^{12} cells/L	9.7	8.95
HGB	g/L	154	137
HCT	%	40.8	38.8
MCV	fL	37.4	40.7
MCH	pg	15.8	15.4
MCHC	g/L	424	378
RDW	%	14	14.4
PLT	10^9 cells/L	464	465
MPV	fL	6.0	5.7
PDW		14.6	14.7

Supplementary Fig. 3 *In vivo* toxicity evaluation of the combination of benzydamine and doxycycline.

CD-1 female mice (n = 6 per group) were gavaged with vehicle or the bezydamine-doxycycline combination once daily for six days. Meanwhile, the mice body weight **(a)**, serum biochemical analysis **(b)** and whole-blood cell analysis **(c)** were shown. The data were presented as mean.

White blood cell (WBC), lymphocyte (LYM), monocyte (MON), neutrophils (NEU), red blood cell (RBC), hemoglobin (HGB), hematocrit (HCT = RBC%), the mean corpuscular volume (MCV, average volume of red cells), mean corpuscular hemoglobin (MCH), platelet count (PLT), and mean corpuscular hemoglobin concentration (MCHC, the average amount of hemoglobin inside a single red blood cell).

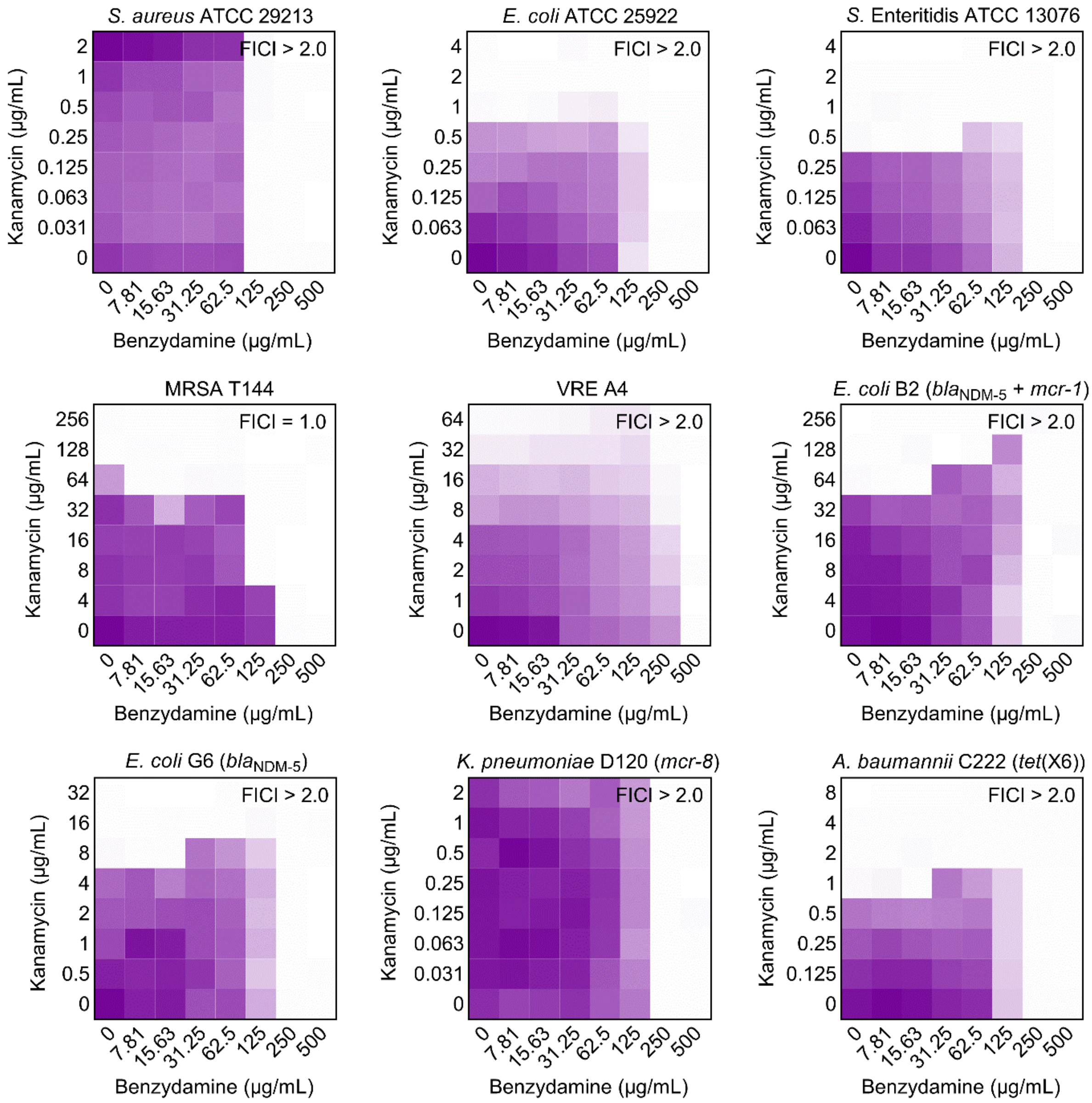

Supplementary Fig. 4 Antagonism effect of benzydamine in combination with kanamycin in both kanamycin-susceptible and -resistant bacteria.

Dark purple represents greater growth. Data represent the mean OD (600 nm) of two biological replicates.

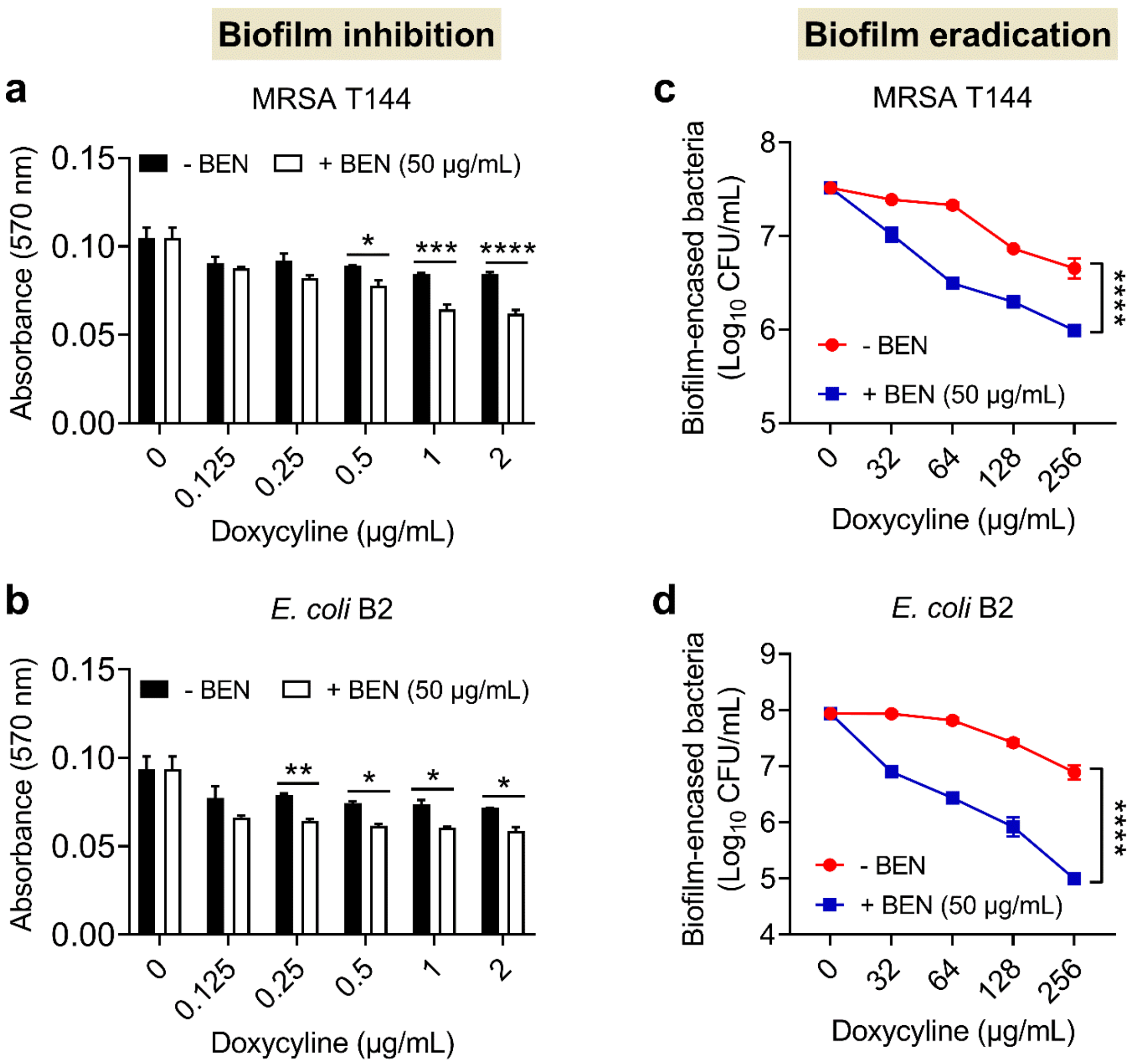

Supplementary Fig. 5 Benzydamine enhances the biofilm inhibition and eradication activities of doxycycline.

(a and b) Benzydamine supplementation potentiates the inhibitory effect of doxycycline on MRSA T144 (a) and *E. coli* B2 (b) biofilm formation.

(c and d) Addition of benzydamine drastically promotes the eradication of established biofilm of MRSA T144 (c) and *E. coli* B2 (d) by doxycycline.

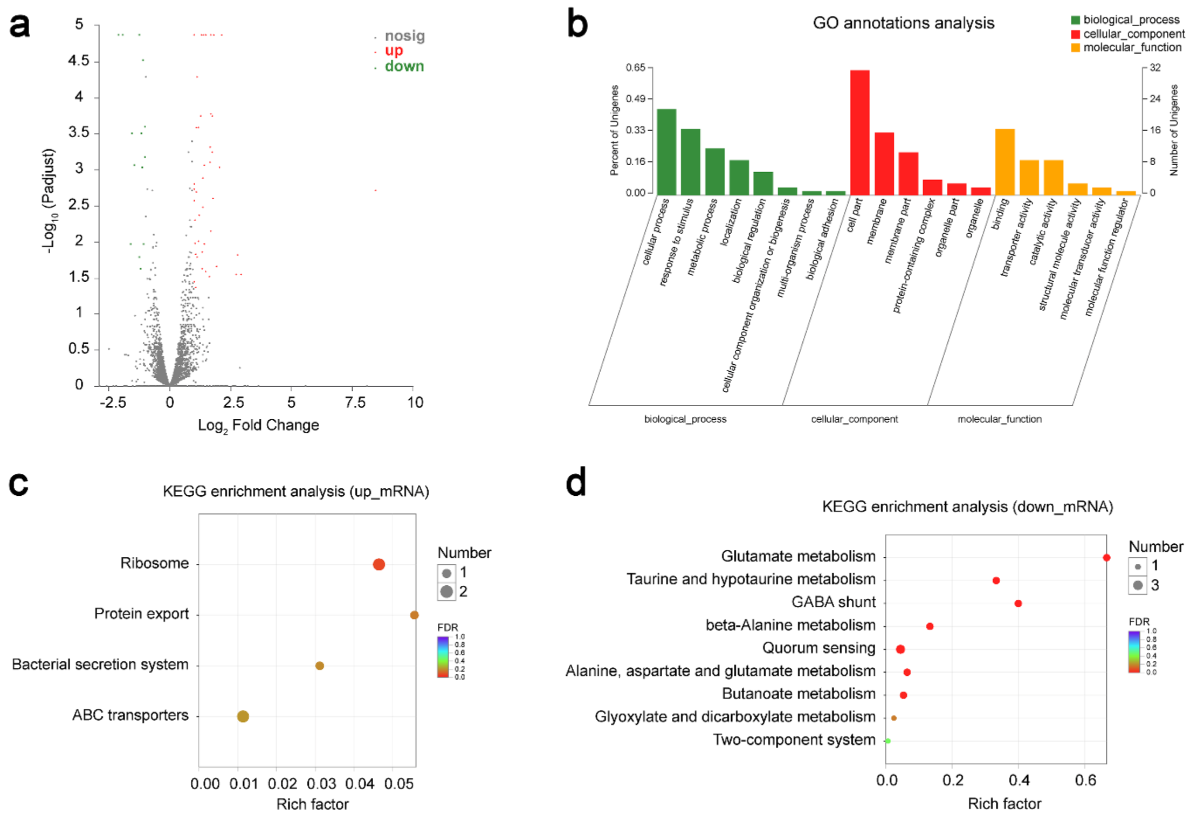

Supplementary Fig. 6 Transcriptomic analysis of *E. coli* B2 after exposure to doxycycline or in combination with benzydamine.

Volcano plot **(a)** and GO (gene ontology) annotation analysis **(b)** of the differential expression genes (DEGs) in *E. coli* B2 after exposing doxycycline (32 µg/mL) or the combination of doxycycline (32 µg/mL) plus benzydamine (250 µg/mL) for 4 h. The x- and y-axes in (a) represent the expression changes and corresponding statistically significant degree, respectively. An adjusted p-value < 0.05 (Student's t-test with Benjamini–Hochberg false discovery rate adjustment) and |log2 Fold change| ≥1 was applied as the cutoff for significant DEGs. KEGG (Kyoto Encyclopedia of Genes and Genomes) enrichment analysis of upregulated DEGs **(c)** and downregulated DEGs **(d)**. The 10 most significant enriched pathways are shown.

Tables

Supplementary Table 1 Bacterial strains used in this study.

Organisms and genotypes	Source/Reference
**Gram-positive bacteria**	
Staphylococcus aureus 29213	ATCC
S. aureus 215 (LZD^R + *cfr*)	[1]
MRSA T144	[1]
Enterococcus faecalis VRE A4	[1]
**Gram-negative bacteria**	
Escherichia coli ATCC 25922	ATCC
E. coli B2 (*mcr-1* + bla_{NDM-5})	In this study
E. coli G6 (bla_{NDM-5})	[1]
Salmonella enteritidis ATCC 13076	ATCC
Klebsiella pneumoniae D120 (*mcr-8*)	In this study
Acinetobacter baumannii C222 (*tet*(X6))	In this study

ATCC, American Type Culture Collection; LZD^R, linezolid resistance.

[1] Y. Liu, Y. Jia, K. Yang, R. Li, X. Xiao, K. Zhu, Z. Wang, *Adv. Sci.* **2020**, *7*, 1902227.

Supplementary Table 2 Synergistic activity of benzydamine and antibiotics against MDR *E. coli* B2.

Antibiotics	MIC[a] (μg/mL)	FIC index	MIC[b] (μg/mL)	Potentiation (fold)[c]
Ampicillin	>128	2.0	>128	–
Meropenem	64	2.0	64	–
Rifampicin	128	1.5	64	2
Vancomycin	128	2.0	128	–
Kanamycin	256	>2	>256	–
Colistin	8	0.5	2	4
Ciprofloxacin	16	0.375	4	4
Doxycycline	32	0.188	2	16
Tetracycline	128	0.25	16	8
Oxytetracycline	256	0.375	64	4
Minocycline	16	0.188	1	16
Tigecycline	2	1.0	1	2

[a/b]MICs of antibiotic in the absence or presence of 0.25×MIC of benzydamine.

[c]Degree of antibiotic potentiation in the presence of 0.25×MIC of benzydamine.

–, none of potentiation activity.

Supplementary Table 3 Synergistic activity of benzydamine and doxycycline against drug-susceptible or -resistant bacteria.

Pathogens	MIC[a] (μg/mL)	FIC index	MIC[b] (μg/mL)	Potentiation (fold)[c]
**Susceptible bacteria**				
S. aureus ATCC 29213	0.5	0.5	0.125	4
E. coli ATCC 25922	1	0.5	0.25	4
S. enteritidis ATCC 13076	2	0.5	0.5	4
E. coli MG1655	2	0.5	0.5	4
**Resistant bacteria**				
MRSA T144	16	0.188	1	16
VRE A4	32	0.375	8	4
E. coli G6	16	0.375	2	8
K. pneumoniae D120	32	0.375	4	8
A. baumannii C222	16	0.5	4	4

[a/b]MICs of antibiotic in the absence or presence of 0.25×MIC of benzydamine.
[c]Degree of antibiotic potentiation in the presence of 0.25×MIC of benzydamine.
–, none of potentiation activity.
