# Supplementary material for: Reversion of antibiotic resistance in multidrug-resistant pathogens using non-antibiotic pharmaceutical benzydamine
Source: Commun Biol. 2021 Nov 25;4:1328. doi: 10.1038/s42003-021-02854-z (PMC8616900; doi:10.1038/s42003-021-02854-z)
Supplement: Supplementary file 2 — Description of Additional Supplementary Files [file 42003_2021_2854_MOESM2_ESM.pdf]

Descriptions of additional supplementary files

Supplementary Data 1 contains the source data for Figure 2, Figure 3, Figure 5, and Figure 6.
